# Elevated PRC1 in gastric carcinoma exerts oncogenic function and is targeted by piperlongumine in a p53‐dependent manner

**DOI:** 10.1111/jcmm.13063

**Published:** 2017-02-12

**Authors:** Bin Zhang, Xiaoting Shi, Guifang Xu, Wei Kang, Weijie Zhang, Shu Zhang, Yu Cao, Liping Qian, Ping Zhan, Hongli Yan, Ka Fai To, Lei Wang, Xiaoping Zou

**Affiliations:** ^1^ Department of GastroenterologyMedical SchoolThe Affiliated Drum Tower Hospital of Nanjing UniversityNanjingJiangsuChina; ^2^ Department of Anatomical and Cellular PathologyState Key Laboratory of Oncology in South ChinaInstitute of Digestive DiseasePartner State Key Laboratory of Digestive DiseasePrince of Wales HospitalThe Chinese University of Hong KongHong Kong SARChina; ^3^ Department of General SurgeryMedical SchoolThe Affiliated Drum Tower Hospital of Nanjing UniversityNanjingJiangsuChina; ^4^ Centre for Experimental AnimalMedical SchoolThe Affiliated Drum Tower Hospital of Nanjing UniversityNanjingJiangsuChina; ^5^ Department of Respiratory MedicineJinling HospitalMedical SchoolNanjing UniversityNanjingJiangsuChina; ^6^ Department of Laboratory MedicineChanghai HospitalThe Second Military Medical UniversityShanghaiChina

**Keywords:** PRC1, gastric cancer, oncogene, piperlongumine, p53

## Abstract

Gastric carcinoma is one of the most common malignancies worldwide and the second most frequent cause of cancer‐related death in China. Protein regulator of cytokinesis 1 (PRC1) is involved in cytokinesis and plays key roles in microtubule organization in eukaryotes. This study was aimed to analyse the expression and to investigate the functional role of PRC1 in gastric tumorigenesis. The expression of PRC1 was evaluated by qRT‐PCR, Western blot and immunohistochemistry. The biological function of PRC1 was determined by CCK‐8 proliferation assays, monolayer colony formation, xenografted nude mice and cell invasion assays by shRNA‐mediated knockdown in AGS and HGC27 cells. The regulation of PRC1 expression by piperlongumine was also investigated using dual‐luciferase reporter assay and ChIP‐qPCR analysis. PRC1 was up‐regulated in primary gastric cancers. Overexpression of PRC1 in gastric cancers was associated with poor disease‐specific survival and overall survival. PRC1 knockdown in AGS and HGC27 cell lines suppressed proliferation, reduced monolayer colony formation, inhibited cell invasion and migration ability and induced cell‐cycle arrest and apoptosis. Inhibition of PRC1 also suppressed tumour growth *in vivo*. We finally confirmed that PRC1 is a novel downstream target of piperlongumine in gastric cancer. Our findings supported the oncogenic role of PRC1 in gastric carcinogenesis. PRC1 might serve as a prognostic biomarker and potential therapeutic target for gastric carcinoma.

## Background

Although the incidence and mortality rates of gastric carcinoma have declined in the west countries since the middle of the 20th century, it remains one of the most common public health problems throughout the world and is still a leading cause of global cancer mortality with an overall 5‐year survival rate of approximately 20% 1, 2. In China, gastric carcinoma is the second most common types of cancers among the male and the third most common types of cancers among the female. It is currently the second leading causes of cancer death among both men and women 3. The compelling evidence has indicated that a progression into the aggressive gastric carcinoma is obviously a multi‐step process with numerous genetic and/or epigenetic alterations. These alterations affect the downstream signal transduction pathways involved in cell proliferation and/or survival, whereas the underlying molecular mechanism is largely unknown 4. Similar to other malignancies, gastric carcinoma is a very heterogeneous cancer type and has traditionally been classified into intestinal and diffuse types according to the Lauren classification 5. Recently, The Cancer Genome Atlas (TCGA) network, classified gastric cancer into four subtypes: Epstein–Barr virus (EBV)‐positive tumours, microsatellite instable (MSI) tumours, genomically stable (GS) tumours and tumours with chromosomal instability (CIN) 6. Trastuzumab and ramucirumab (targeting HER2 and VEGFR, respectively) are currently the only gastric cancer‐approved targeted therapies. Given that the use of targeted therapies in gastric tumours is much less common than in other cancers, the newly defined molecular classification of gastric cancer may represent a critical starting point to design more appropriate clinical trials based on the principles of precision medicine.

A hallmark of cancers is unlimited cellular proliferation; proper control of entry into and progression through the cell growth and division cycle is essential for cell proliferation and the maintenance of genome stability 7. To do so, cells must precisely duplicate their chromosomal DNA during S phase, segregate the sister chromatids to opposite poles of the mitotic spindle during mitosis, assemble two nuclei and then divide their organelles, cytoplasm, plasma membrane and other cell contents into two daughter cells in the process known as cytokinesis, which is the final stage of the cell cycle 8. The PRC1, also known as Ase1(yeast)/MAP65 (plant) gene was originally identified as a CDK substrate in an *in vitro* phosphorylation screen and was subsequently found to be a mid‐zone‐associated protein required for cytokinesis 9. PRC1 is phosphorylated by CDK1 (Cdc2/cyclin B) in early mitosis and turns into an inactive and monomeric state 10. During the metaphase–anaphase transition, it is dephosphorylated and interacts with KIF4, a kinesin motor that translocates PRC1 along mitotic spindles towards the plus end of antiparallel interdigitating microtubules. The dephosphorylated PRC1 protein bundles the antiparallel interdigitating microtubules to establish the mid‐zone that is necessary for cytokinesis 11.

In addition to its fundamental role in cytokinesis, accumulating evidence also suggests that PRC1 appears to be linked with human carcinogenesis. PRC1 is overexpressed in a variety of cancers, including breast cancer 12, bladder cancer 13, hepatocellular carcinoma 14, 15 and pancreatic cancer 16. Knockdown of PRC1 using siRNA significantly suppresses the growth of breast and bladder cancer cells, indicating its crucial role in proliferation of cancer cells, and also suggesting PRC1 is a promising molecular target for human cancer treatment 12, 13. To date, however, the impact of PRC1 expression on gastric carcinoma patient survival and its potential oncogenic role and molecular mechanisms in gastric carcinoma has not been elucidated. In this study, we studied PRC1 expression status and its clinical significance in gastric carcinoma. Both *in vitro* and *in vivo* functional assays were performed to characterize the biological effects of PRC1 in gastric carcinoma. More importantly, we demonstrate, for the first time, that PRC1 can be targeted by piperlongumine (PL), an agent that has been previously proved to suppress gastric cancer cells by our group 17, *via* a p53‐dependent mechanism. Our findings shown in this study suggest that PRC1 might play critical roles in tumour cell growth and be a promising target for the development of novel anticancer drugs to gastric carcinoma.

## Materials and methods

### Gastric cancer cell lines and clinical samples

Human gastric cancer cell lines AGS and HGC27 were purchased from American Type Culture Collection of the Chinese Academy of Sciences (Shanghai, China) and were cultured in RPMI 1640 (Wisent Biotec, Co. Ltd. Montreal, QC, Canada) containing 10% foetal bovine serum (Wisent Biotec, Co. Ltd) in a humidified 5% CO_2_ atmosphere at 37°C. A total of 17 primary gastric carcinomas and their paired non‐cancerous gastric mucosal tissues were obtained from patients who underwent curative surgery in 2013 at the Department of Gastrointestinal Surgery (Nanjing Drum Tower Hospital, China) after obtaining written informed consent. All specimens were immediately snapped‐frozen in liquid nitrogen and stored at −80°C until processing. Archival tissue blocks from 133 patients with gastric adenocarcinoma were retrieved from the Department of Anatomical and Cellular Pathology, Prince of Wales Hospital, the Chinese University of Hong Kong and arranged in tissue array blocks and have been described elsewhere 18, 19, 20. All experiments were conducted and approved in accordance with the guidelines of ethics committees of Nanjing University and the Chinese University of Hong Kong.

### Reagents, plasmids and antibodies

FITC‐Phalloidin, 4′, 6‐diamidino‐2‐phenylindole (DAPI), PL, SML0221 and PKH67 Fluorescent Cell Linker Kits were from Sigma‐Aldrich (St. Louis, MO, USA). Lentivirus plasmid vectors pLKO.1‐puro vectors containing non‐targeting shRNA (CAACAAGATGAAGAGCACCAA) and shRNA targeting PRC1 (shPRC1#1, CCTGAAGGAAAGACTCATCAA and shPRC1#2, CAGGAACATTCAAAGGCATTT) were purchased from Sigma‐Aldrich. Promoterless (pGL3 basic), SV40 promoter‐driven (pGL3‐SV40) and pRL‐TK luciferase reporter vector were purchased from Promega (Madison, WI, USA). The full‐length PRC1 promoter reporter plasmid was a kind gift from Dr.Liu Jingwen 21. The resultant promoter reporter plasmids were generated by inserting the serial deleted fragments of the 5‐flanking region of PRC1 promoter upstream of the initiating ATG into pGL3‐basic vector digested with KpnI and XhoI in the sense orientation. p53 expression vector (pcDNA3.1‐p53) was constructed by Dr. Thomas Roberts 22, and empty vector (pcDNA3.1) was purchased from Addgene. siRNAs against p53 (ONTARGETplus SMARTpool Tp53 siRNA) and the non‐targeting siRNAs (Control‐siRNA) were purchased from GE Healthcare Dharmacon Inc.(Lafayette, CO, USA). PRC1 (sc‐8356), PARP (sc‐7150), GADD45α (sc‐797) and p53 (sc‐6243) antibodies were from Santa Cruz Biotechnology (Santa Cruz, CA, USA). Caspase‐9 (#9502), caspase‐3 (#9662), cleaved PARP (#5625), CDK1 (#9116), CDK2 (#2546), CDK6 (#13331), cyclin B1 (#4138), cyclin E1 (#4129) and p27 (#3686) antibodies were from Cell Signaling Technology (Beverly, MA, USA). p53 (ab28) antibody was from Abcam. β‐Actin (#A5441) antibody was from Sigma‐Aldrich.

### Lentivirus production and transduction

Lentivirus expressing shPRC1#1, shPRC1#2 or shControl was produced as described previously 23. Cells were transduced with appropriated amount of lentivirus for 24 hrs in the presence of polybrene (5 μg/ml). Then, the supernant was removed and replace with fresh medium. The cells were cultured for 4–6 days and then harvested for the experiments.

### Cell proliferation assay

Cell proliferation in response to PRC1‐silencing was measured by Cell Counting Kit‐8, according to manufacturer's instructions (Dojindo, Kumamoto, Japan). Cells were seeded in 96‐well plates after transduction of lentivirus in 6‐well plates for 48 hrs. The result was expressed as the maximum absorbance at 450 nm, based on three independent experiments.

### Colony formation assay

Cells were seeded into new 6‐well plates (1000 cells per well) after transduction of lentivirus in 6‐well plates for 2 days and incubated with medium containing 5 μg/ml of puromycin for 14 days. Colonies were fixed with methanol for 15 min. and stained with 0.5% crystal violet for 20 min. at room temperature sequentially. The number of colonies with more than 50 cells was counted manually under a microscope. Experiments were performed in triplicate.

### RNA extraction and Quantitative real‐time PCR

Total RNA in tissue specimens or cells was isolated using TRIzol reagent, and reverse transcription was carried out with 500 ng RNA in a total 10 μl reaction volume using PrimeScript™ RT Master Mix (TaKaRa, Shuzo Co., Ltd., Kyoto, Japan) according to the manufacturer's instructions. Quantitative real‐time PCR experiments were carried out with the Step‐One PCR System (Applied Biosystems, Foster City, CA). The sequences of the amplification primers are listed in Table S1. Real‐time RT‐PCR data analysis was conducted according to the 2^−ΔΔCt^ methods using the threshold cycle (Ct) values for target genes and β‐actin, as an endogenous control gene. All assays were performed in triplicate.

### Western blot

Western blotting was performed as previously described 24, 25. Proteins were resolved by SDS–polyacrylamide electrophoresis and were transferred to a nitrocellulose membrane and immunoblotted with antibodies as indicated, followed by appropriate HRP‐conjugated secondary antibodies. Signals generated by enhanced chemiluminescence (Millipore, Billerica, MA, USA) were recorded with a CCD camera (CLINX, Shanghai, China). Data are representative of at least three independent experiments.

### Migration and invasion assays

Cell migratory and invasive abilities were assessed by way of transwell (Corning Life Sciences, Bedford, MA, USA) and Matrigel invasion (BD Biosciences, San Jose, CA, USA), respectively, which has been described previously 26. Briefly, cells after 3 days of transduction were resuspended and cultured in medium containing only 1% FBS in the upper compartment of the chamber, whereas regular medium containing 10% FBS was added into the lower compartment of chambers. For transwell migration assay, 5 × 10^4^ cells/well were seeded, whereas 8 × 10^4^ cells/well were seeded for the invasion assay. After 24 hrs, the cells on the topside of the membrane were scraped using cotton swab, and the cells that migrated to the bottom were fixed with methanol and stained with 0.5% crystal violet. The migrated or invaded cells were counted in five sequential visual fields under 400‐fold magnification. Each experiment was performed in replicate inserts, and mean value was calculated from three independent experiments.

### Cell‐cycle and apoptosis analysis

For cell‐cycle analysis, cells were synchronized with serum starvation for 24 hrs and then transduced with lentivirus on 6‐well plates for 4 days. Cell‐cycle analysis was conducted using BD Cycle test™ plus DNA Reagent Kit (340242; BD Biosciences). Cell‐cycle distribution was determined using a flow cytometer (BD FACSAria™ II, San Jose, CA, USA). Apoptotic cell death was measured by Annexin V fluorescein isothiocyanate (FITC) Apoptosis Detection Kit I (556547; BD Biosciences) according to the manufacturer's instructions. AGS and HGC27 cells transduced with indicated shRNAs were collected and washed with cold PBS twice and then were resuspended in 100 μl of Annexin V binding buffer and incubated with 5 μl of FITC‐conjugated Annexin V and 5 μl of propidium iodide for 15 min. in the dark. Annexin V binding buffer (400 μl) was then added. Finally, the cells were analysed by BD FACSCanto II flow cytometer (BD Biosciences). The results were obtained from at least three independent experiments with triplicated wells each time.

### Phalloidin and PKH67 staining

Phalloidin staining was conducted as previously described 26. Cells transduced with lentivirus were replated on glass coverslips. Cells were first fixed in 4% ice‐cold paraformaldehyde for 15 min. on ice. Subsequently, fixed cells were blocked with 1% bovine serum albumin (BSA)/PBS for 1 hrs. FITC‐Phalloidin (5 μg/ml, Sigma‐Aldrich) was then used to stain filamentous actins for 2 hrs at room temperature. Fluorescence was observed using an inverted fluorescence microscope from Carl Zeiss (Axio Observer A1; Zeiss, Germany). PKH67 dye was used for cell membrane labelling. According to the manufacturer's instructions, the cells were rinsed with serum‐free medium once. Dilution C was used to resuspend the cells, with PKH67 dye (2 μg/ml) added, and the cells were subcultured for three generations. Residual fluorescence was observed using an inverted fluorescence microscope from Carl Zeiss as mentioned above.

### DNA transfection and luciferase reporter assay

DNA transfection and luciferase reporter assay were performed as previously described 26. Cells were plated to 24‐well plates 24 hrs prior to transfection experiments. Transfection experiment was carried out by adding 380 ng of reporter plasmid along with a pRL‐TK reporter plasmid (20 ng) as a control for transfection efficiency. All assays were performed using a Dual Luciferase Reporter Assay System (Promega).

### Chromatin immunoprecipitation

Chromatin immunoprecipitation (ChIP) assay was conducted as previously described 25. Primers used in ChIP‐qPCR experiments are shown in Table S2. Occupancy of p53 protein was normalized to input and expressed as a percentage of the input DNA (% Input). Data were obtained from at least three independent experiments.

### Tumour xenograft model in nude mice

All animal procedures and care were approved by the Institutional Animal Care and Use Committee of Nanjing Drum Tower Hospital, Medical School of Nanjing University. HGC‐27 cells (~3 × 10^6^ cells/mice) transduced with indicated lentiviral shRNAs were resuspended in 100 μl serum‐free medium and gently mixed with the same volume of ice‐cold Matrigel and were injected subcutaneously into the right flank of 4‐ to 6‐week‐old female BALB/c nu/nu nude mice (*n* = 5). Tumour size was measured and calculated as previously described 27. Tumour volume and animal weight were measured every 3 days. Mice were killed when control group tumours reached ~1000 mm^3^. Tumours were then excised and weighed.

### Statistical analysis

Data were analysed with graphpad prism 6.00 (GraphPad Software, La Jolla, CA, USA). Data were expressed as the means ± S.E.M. of at least three independent experiments. Statistically significant differences between the experimental and control groups were identified by Student's *t*‐test, ordinary one‐way anova, and two‐way anova followed by Dunnett's multiple comparisons test. Differences were considered statistically significant when *P* < 0.05.

## Results

### PRC1 is aberrantly expressed in gastric carcinoma and correlates with patients’ survival

**Figure 1:**
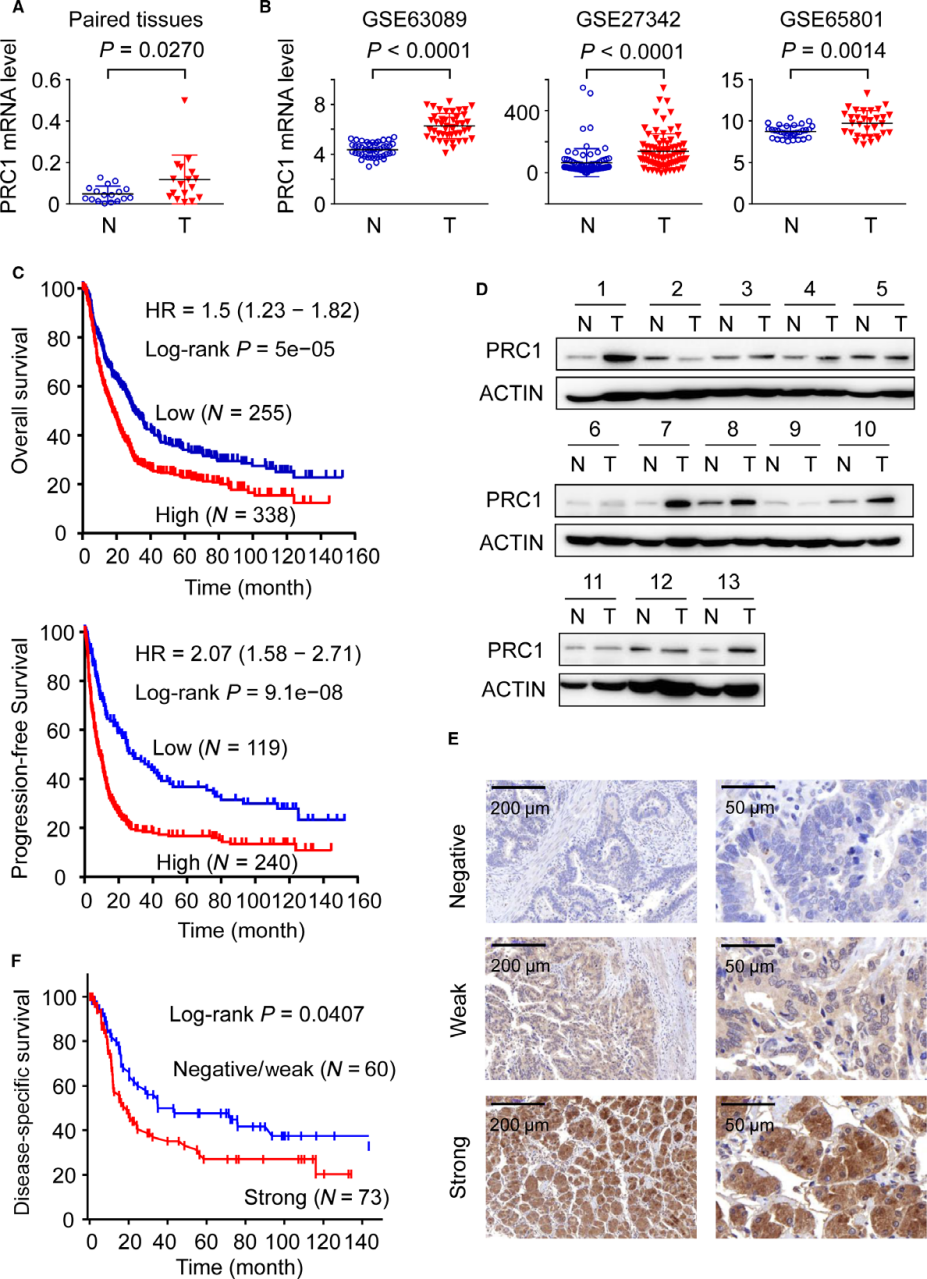
PRC1 overexpression in gastric cancers and its association with patients’ survival. (**A**)Recapitulated is the gene expression of PRC1 mRNA in gastric cancer tissues (T) and adjacent non‐tumoural tissues (N) based on quantitative real‐time analysis of paired specimens (*N* = 17). *P* values were generated using the paired Student's *t*‐test. (**B**) PRC1 gene expressions in gastric cancer tissues (T) and adjacent non‐cancerous tissues (AN) as determined by gene expression array. Data were from NCBI, GEO database GSE63089 (*N* = 45), GSE27342 (*N* = 80) and GSE65801 (*N* = 32), respectively. *P* values were generated using the unpaired Student's *t*‐test. (**C**) Kaplan–Meier plot showing overall survival and time to first progression of gastric cancer patients stratified by high or low PRC1 mRNA expression. These data are from 593 gastric tumour samples using publicly available data sets (http://kmplot.com/analysis/index.php?p=service&cancer=gastric). (**D**) Western blot analysis of PRC1 in a subset of paired gastric cancer tissues. β‐Actin was used as the endogenous loading control. N, non‐tumour; T, tumour. (**E**) Representative immunohistochemistry (IHC) staining for PRC1 expression in gastric cancer tissues. Scale bar, 200 μm for left panel and 50 μm for right panel, respectively. (**F**) Kaplan–Meier plot showing disease‐specific survival curve according to PRC1 staining status in gastric adenocarcinoma.

We first determined and compared PRC1 mRNA expression of 17 pairs of samples from patients who underwent resection of primary gastric carcinoma using qRT‐PCR. As shown in Figure 1A, mRNA level of PRC1 gene is markedly increased in gastric carcinomas relative to the paired adjacent non‐tumour tissues. To generalize our finding, we recapitulated PRC1 gene expression from the large cohorts of gastric cancer patients that are available from the GEO database (accession numbers GSE63089, GSE27342 and GSE65801) and data are given as scatterplots. Consistently, PRC1 gene expression was significantly up‐regulated in all three different gastric cancer cohorts (Fig. 1B). We then compared the PRC1 expression level between different subtypes of gastric cancer by analysing the TGGA cohort. With regard to the Lauren classification, PRC1 mRNA level is significantly higher in intestinal type when compared with diffuse type (Fig. S1A). In addition, when dividing the cohort into four molecular subtypes, PRC1 mRNA is abundantly expressed in MSI groups, then CIN and EBV groups. It is relatively lowly expressed in GS group (Fig. S1B). Further analysis revealed that MSI gastric cancers are enriched for the intestinal histological variant (Fig. S1C); thus, further study is warranted to investigate why PRC1 mRNA level is elevated in intestinal type of gastric cancer. Next, computational analysis using the KM plotter integrative bioinformatic interface 28 revealed a significant correlation between high PRC1 mRNA expression and poor overall and disease‐free survival (Fig. 1C). Western blotting showed that up‐regulation of PRC1 protein was detected in 7 of 13 (53.8%) randomly selected gastric carcinomas. Statistical analysis revealed that gastric cancer tissues expressed a significantly higher level of PRC1 protein than adjacent non‐tumoural tissues (Fig. 1D and Fig. S1D). Immunohistochemistry was then performed to assess the PRC1 protein expression in tissue microarrays containing 133 formalin‐fixed, paraffin‐embedded gastric carcinomas. The cancer cells often exhibited cytoplasmic expression of PRC1 (Fig. 1E). Negative staining of PRC1 was observed in 7 % (10/133) of samples, weak staining was noted in 38% (50/133), and strong immunoreactivity was seen in 55% (73/133) of the samples, respectively. PRC1 expression in immunohistochemistry did not show association with any clinicopathologic parameter (Table S3). More intriguingly, overexpression of PRC1 was correlated significantly with shorter overall survival (Fig. S1E) and shorter disease‐specific survival of patients (Fig. 1F). These results indicated that PRC1 expression was significantly increased in gastric cancer tissues and the up‐regulation may represent a novel marker for patients’ prognosis.

### Short hairpin RNA‐mediated PRC1 silencing abolishes the tumorigenic effect both *in vitro* and *in vivo*

**Figure 2:**
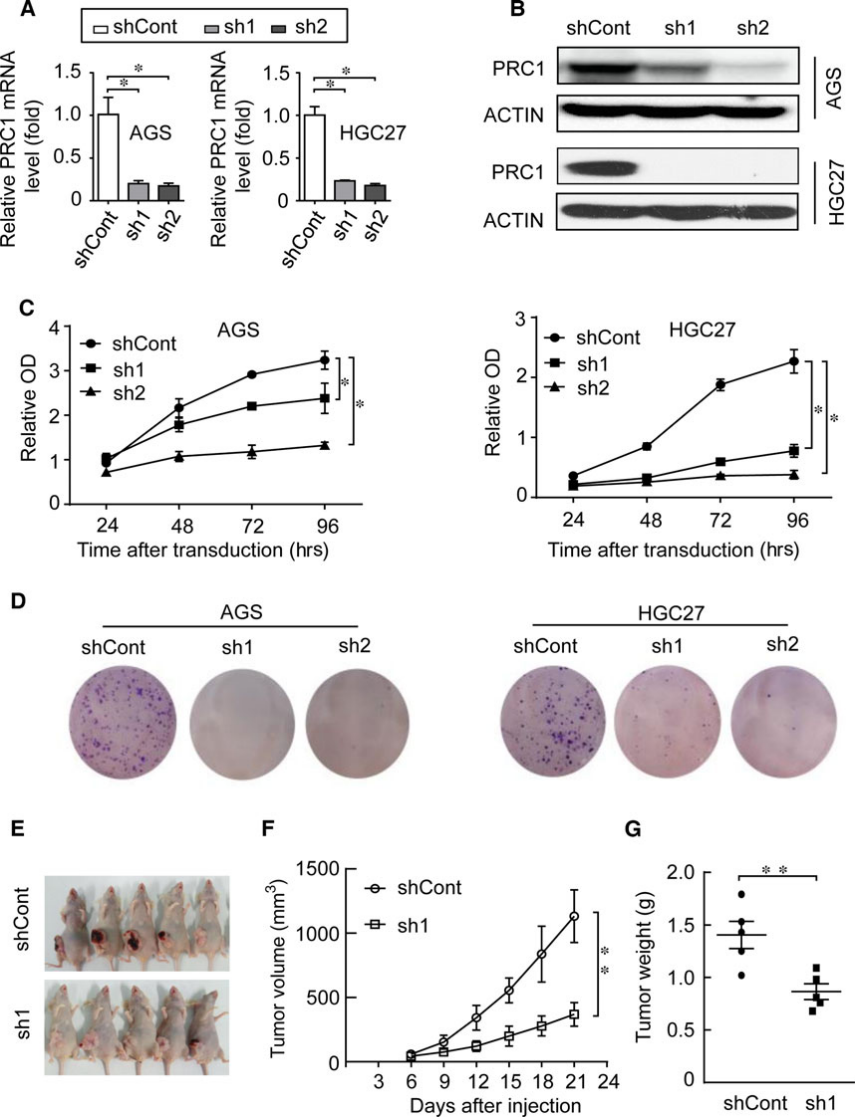
shRNA‐mediated PRC1 silencing suppresses tumour growth both *in vitro* and *in vivo*. (**A**) and (**B**) AGS and HGC27 cells were treated with non‐target control shRNA (shCont) or shRNA against PRC1 (sh1 and sh2). Quantitative real‐time PCR and Western blot analysis were performed to detect PRC1 expression. β‐Actin was used as loading controls.*P < 0.01, Student's *t*‐test. (**C**)The cell growth rates were determined with CCK‐8 proliferation assay. *P < 0.05, Student's *t*‐test. (**D**) Colony formation assay of gastric cells transduced with lentiviruses expressing the indicated shRNA. (**E**) Representative images of the tumours formed in nude mice induced by HGC27 cells transduced with indicated shRNA. (**F**) Mean xenograft tumour volumes were plotted against days after injection. (**G**) Weights of the excised xenograft tumours were summarized. **P < 0.01, Student's *t*‐test. Three independent experiments were conducted in **A**,** C** and **D**, each in triplicate. Values are means ± standard deviation (S.D.) for triplicate determinations from three different cultures. Data obtained from a representative experiment are shown.

We next examined whether PRC1 is required for the tumorigenic phenotypes of gastric cancer cells by silencing PRC1 expression with short hairpin RNA. The introduction of shRNA into the gastric cancer cell lines AGS and HGC27 dramatically decreased the expression level of PRC1 relative to control cells expressing scrambled control shRNA (Fig. 2A and B). Functional assays revealed that PRC1 depletion significantly decreased the cell growth as determined by CCK‐8 assay (Fig. 2C), and colony formation efficiencies as determined by colony formation assay (Fig. 2D) compared with control cells. To further investigate whether PRC1 knockdown suppresses tumour growth *in vivo*, we generated stable PRC1 knockdown HGC27 cell population. When the stable cells were injected into mice subcutaneously, the PRC1‐depleted tumours grew much more slowly than the scramble control tumours did (Fig. 2E and F). The mean tumour weight from PRC1‐depleted cells was less than one‐third of those from scramble control cells after 21‐day growth *in vivo* (Fig. 2G). Collectively, these data indicate that PRC1 is essential for gastric cancer cell proliferation both *in vitro* and *in vivo*.

### Knockdown of PRC1 promotes cell‐cycle arrest and apoptosis

**Figure 3:**
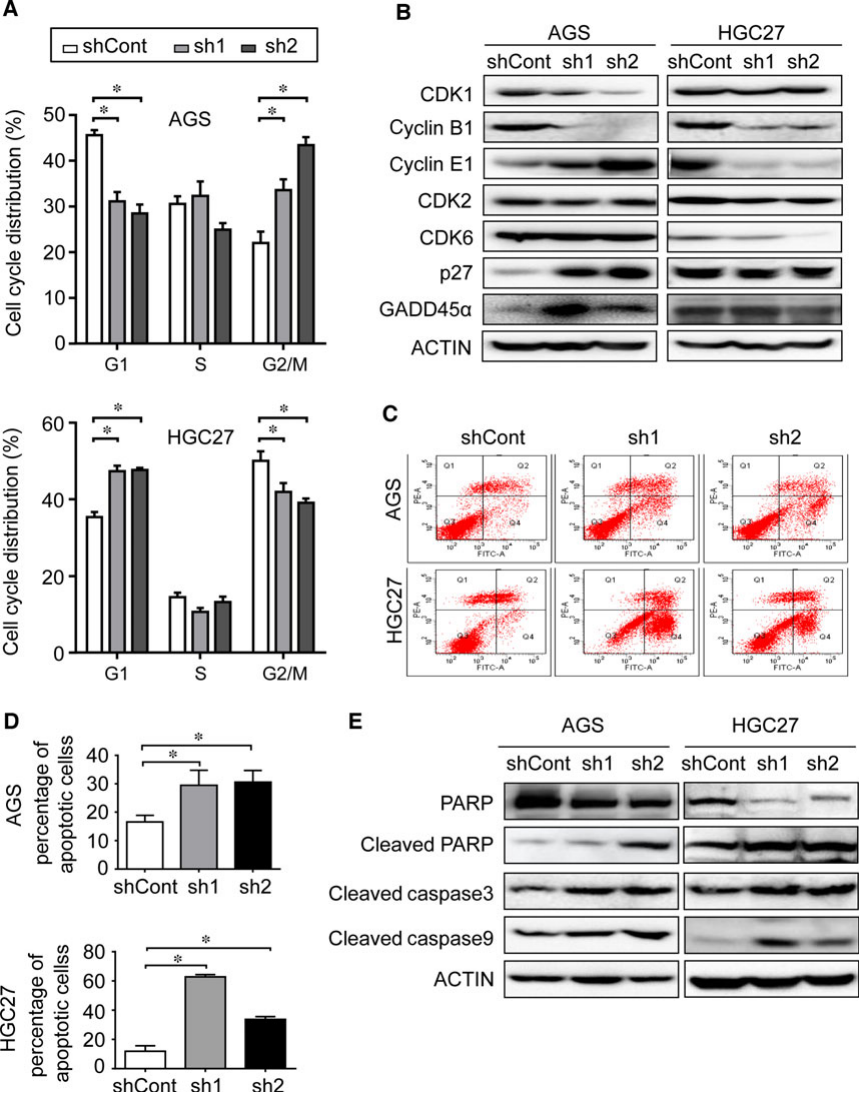
Inhibition of PRC1 leads to cell‐cycle arrest and apoptosis. (**A**) Cell‐cycle analysis of cells transduced indicated shRNA using PI and flow cytometric analysis. *P < 0.01, when compared with shCont. (**B**) Western blot analysis of the protein expression of cell‐cycle regulators. β‐Actin was used as the loading control. (**C**) Flow cytometry analysis of the apoptotic cells using Annexin V/PI double‐staining assay. (**D**) Positive Annexin V cells were displayed as histogram. *P < 0.05, when compared with shCont. (**E**) Western blotting was used to analyse the expression of full‐length and cleaved PARP, cleaved caspase‐3 and cleaved caspase‐9. β‐Actin was used as the loading control. (**A‐D**), all experiments were carried out in triplicate and representative results from three independent experiments are shown. Bar means mean ± S.D. for triplicate determinations from three different cultures.

To further test whether PRC1 depletion regulates the cell cycle, we knocked down PRC1 in AGS and HGC27 cells and then examined the cell cycle using fluorescence‐activated cell sorting (FACS) analysis. As expected, PRC1 depletion in AGS cells significantly increased the percentage of cells in the G2 phase and decreased the percentage of cells in the G1 phase (Fig. 3A, upper). In contrast, PRC1 depletion in HGC27 cells significantly increased the percentage of cells in the G1 phase and decreased the percentage of cells in the G2 phase (Fig. 3A, lower). Thus, it is likely that PRC1 depletion block cell‐cycle progression at different phases. To validate these results and further investigate why PRC1 reduction affects cell‐cycle distribution differently between the two cell lines, we examined the expression of cell‐cycle regulators operative in G1 and G2 phases, including CDK1, CDK2, CDK6, cyclin B1, cyclin E1, p27 and GADD45α. As shown in Figure 3B, when PRC1 was depleted in AGS cells, expression of CDK1 and cyclin B1 was obviously inhibited, whereas when PRC1 was silenced in HGC27 cells, expression of cyclin E1 and CDK6 was obviously reduced. However, no significant changes were observed in the expression of other regulators, including cyclin A and CDK2 (Fig. 3B and data not shown). Furthermore, p27 and GADD45α, which inhibits cell‐cycle progression from G2 to M phase in gastric cancer cells 24, 29, were remarkably increased in AGS cells but not HGC27 cells when PRC1 was depleted (Fig. 3B). These data suggest the discrepancy of cell‐cycle distribution may be due to the selective induction of p27 and GADD45α in gastric cancers following PRC1 depletion. We next investigated the potential apoptotic effect of PRC1 knockdown using the FACS assay. As shown in Figure 3C and D, sh1‐ and sh2‐transduced cells underwent apoptosis much more frequently than the negative control shRNA‐transduced cells. Consistent with this observation, transduction with sh1 and sh2 in cells induced the cleavage of caspase‐9, caspase‐3 and PARP compared with the control shRNA‐transduced cells (Fig. 3E). These results indicate that PRC1 gene silencing inhibits gastric cancer cell proliferation at least partially through induction of cell‐cycle arrest and cellular apoptosis.

### Knockdown of PRC1 impedes gastric cancer cell migration and invasion

**Figure 4:**
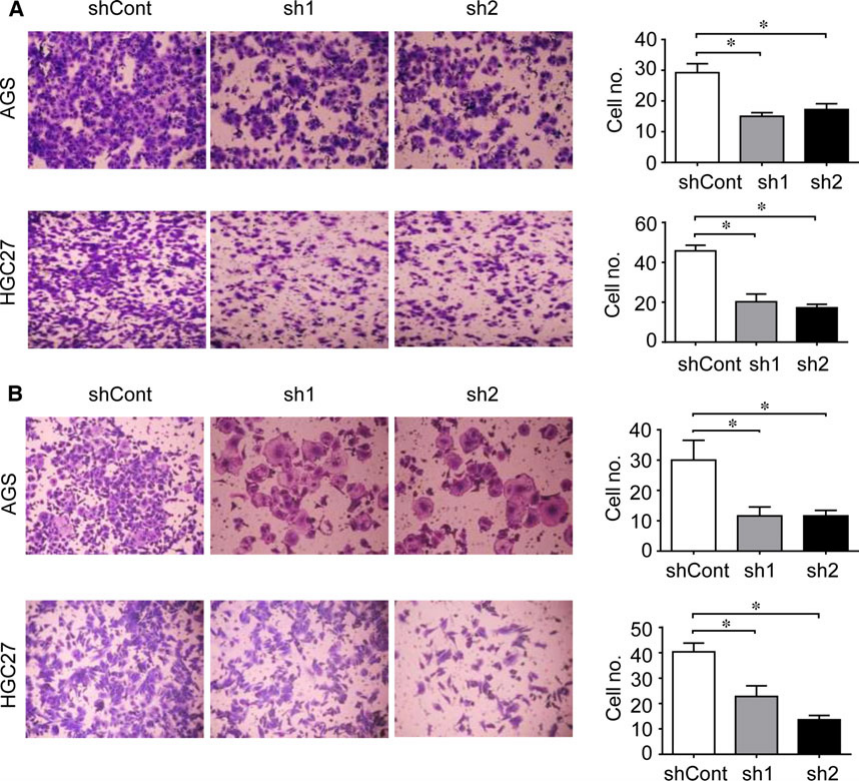
Knockdown of PRC1 impedes gastric cancer cell migration and invasion. Significant reduction in cell migration (**A**) and cell invasion (**B**) was found in cells expressing sh1 and sh2, compared with cells expressing shCont. Representative images of migrated AGS and HGC27 cells in each group are shown in the left. Cells migrated through the pores of transwell plates were counted and reported in the right. Data are presented as mean ± S.D. of three independent experiments. *P < 0.05, when compared with shCont.

Next, we investigated the effect of PRC1 depletion on migratory capacity of gastric cancer cells. Transwell migration assay revealed significantly reduced migrated cells in PRC1‐depleted cells compared with that in control (Fig. 4A). Concordantly, knockdown of PRC1 gene also impaired cell invasion through Matrigel (Fig. 4B). Together, these data suggested a role of PRC1 in the motility and invasiveness of gastric cancer cells.

### Knockdown of PRC1 selectively impairs cytokinesis in AGS but not HGC27 cells

**Figure 5:**
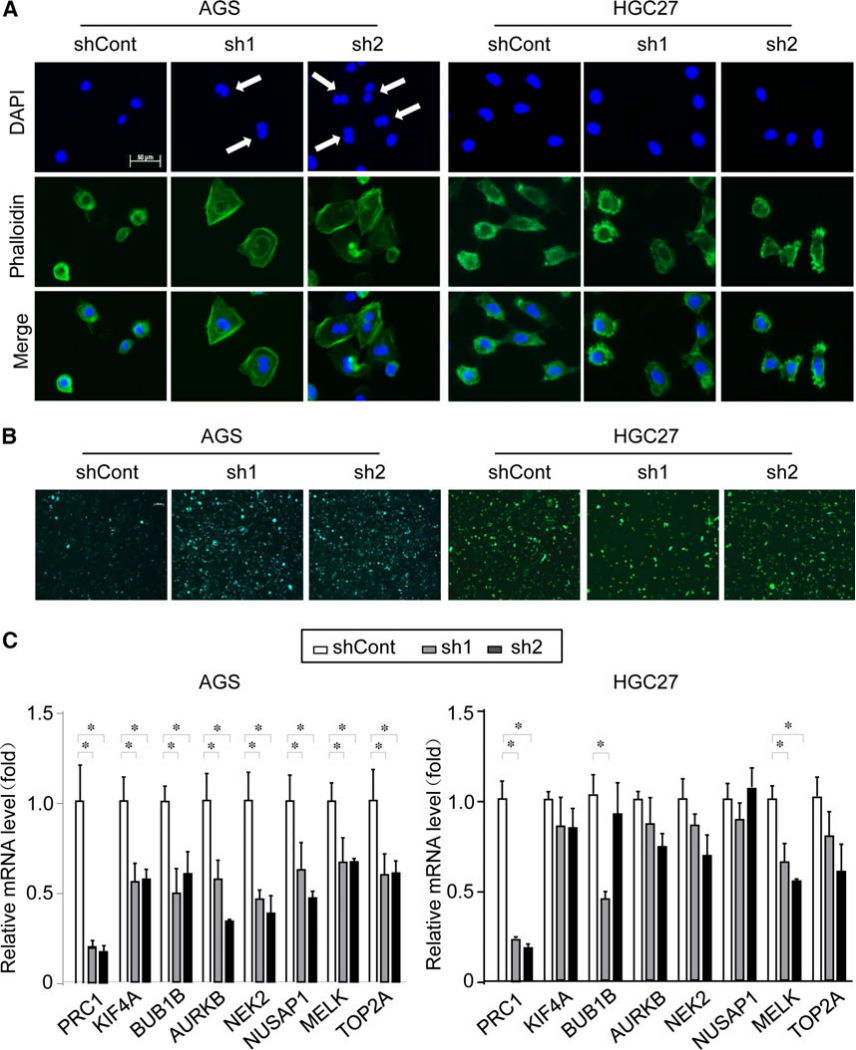
Knockdown of PRC1 selectively impairs cytokinesis in AGS but not HGC27 cells. (**A**) Cells expressing indicated shRNA were analysed using phalloidin (FITC, green) and DAPI (blue). Scale bar, 50 μm. (**B**) The cell membrane was stained using the fluorescent dye PKH67 after continuous subculture for 2 generations. Scale bar, 100 μm. (**C**) Quantitative real‐time analysis of mRNA levels of genes related to mitosis. β‐Actin mRNA expression was used as an internal control. Experiment was conducted in triplicate. Values represent the mean ± S.D. of 3 independent experiments. *P < 0.01, when compared with shCont.

Flow cytometry demonstrated that AGS cells transduced with PRC1 shRNAs contained a significantly larger number of tetraploid cells (Fig. S2), indicating that PRC1 genetic silencing impaired the elaborate co‐ordination of nuclear division and cytokinesis. Consistently, fluorescence assay revealed that AGS cells expressing PRC1 shRNAs exhibited increased nuclear abnormality characterized by multi‐nuclei formation, whereas HGC27 cells expressing PRC1 shRNAs exhibited no obvious nuclear abnormality compared with the control cells (Fig. 5A). To further identify whether PRC1 depletion blocked cytokinesis in AGS but not HGC27 cells, cell membrane was stained using the fluorescent dye PKH67. After continuous subculture for two generations, fluorescent microscopy found that the AGS cells but not HGC27 cells transduced with PRC1 shRNAs contained significantly more residual PKH67 dye than the control group (Fig. 5B), indicating that PRC1 depletion selectively postponed cytokinesis in AGS cells. We next sought to determine the underlying mechanism accounting for the differential role of PRC1 on cytokinesis. Our data further showed that PRC1 depletion probably disrupted mitotic checkpoint signalling because genes involved in mitotic arrest, including KIF4A, BUB1B, AURKB, NEK2, NUSAP1, MELK and TOP2A, were commonly repressed on PRC1 knockdown in AGS cells instead of HGC27 cells (Fig. 5C).

### Piperlongumine inhibits PRC1 expression *via* a p53‐dependent mechanism

**Figure 6:**
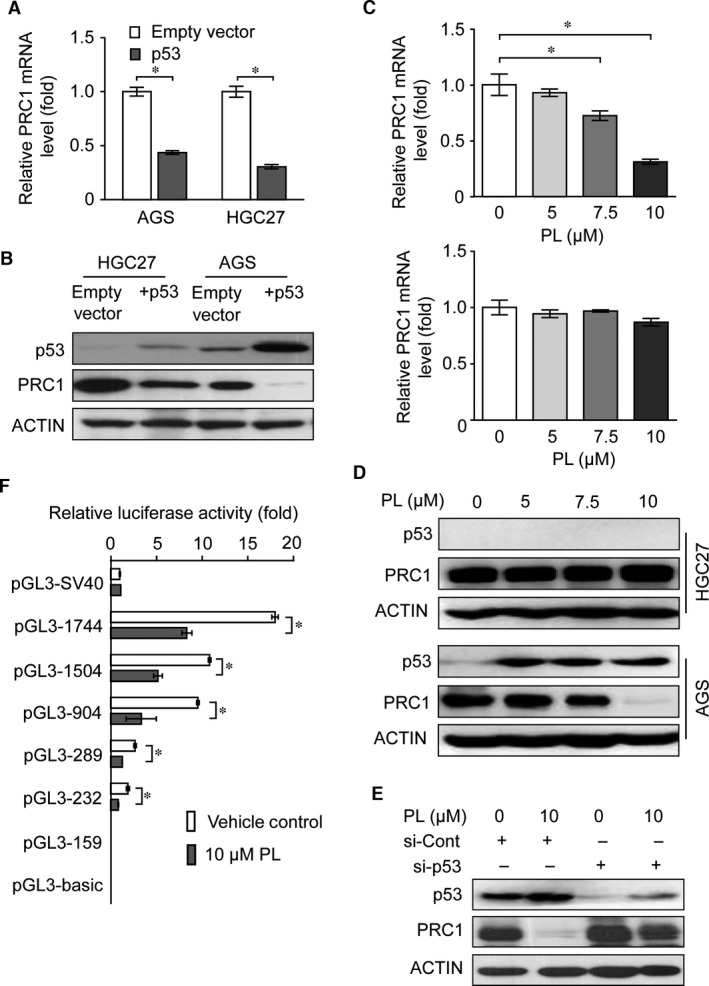
Piperlongumine inhibits PRC1 expression *via* a p53‐dependent mechanism. (**A**) Quantitative real‐time analysis of PRC1 mRNA level in AGS and HGC27 cells transfected with p53‐expressing plasmid or empty vector. β‐Actin mRNA expression was used as an internal control. Experiment was conducted in triplicate. Values represent the mean ± S.D. of three independent experiments. *P < 0.01, when compared with cells transfected with empty vector. (**B**) Western blotting analysis of p53 and PRC1 proteins in AGS and HGC27 cells transfected with p53‐expressing plasmid or empty vector. β‐Actin was used as the loading control. (**C**) Quantitative real‐time analysis of PRC1 mRNA level in AGS and HGC27 cells treated with or without increased concentrations of piperlongumine for 24 hrs. β‐Actin mRNA expression was used as an internal control. Experiment was conducted in triplicate. Values represent the mean ± S.D. of three independent experiments. *P < 0.01, when compared with cells treated with vehicle control. (**D**) Western blotting analysis of p53 and PRC1 in AGS and HGC27 cells treated with various concentrations of piperlongumine for 24 hrs. β‐Actin was used as the loading control. (**E**) AGS cells were transfected with either negative control‐siRNA or p53‐specific siRNA for 48 hrs and then followed by treatment of 10 μM of piperlongumine for 24 hrs, and whole‐cell lysates were separated by SDS‐PAGE and then reacted with indicated antibodies. (**F**) Normalized luciferase activity of various PRC1 promoter reporters in AGS cells treated with piperlongumine at 10 μM or with vehicle control for 24 hrs. pRL‐TK reporter plasmid was cotransfected as a control for transfection efficiency. The relative activity of pGL3‐SV40, a luciferase reporter gene construct driven by the SV40 promoter in control cells was designated as 1.0. Results are the mean ± S.D. of triplicate measurements. *P < 0.01.

Our recent work has identified that PL induces gastric cancer cell apoptosis and G2/M cell‐cycle arrest both *in vitro* and *in vivo*.^17^ Notably, we have demonstrated that PL induces p53 expression in the p53 wild‐type AGS cells but not in the p53‐mutated HGC27 cells 17. Previously it was reported that p53 directly suppresses PRC1 gene transcription *via* interaction with PRC1 gene promoter in a variety of cancer cells 21. Consistently, our data revealed that ectopic expression of p53 also suppresses PRC1 expression in gastric cancer cells (Fig. 6A and B), indicating PRC1 is a common downstream target of p53. Furthermore, PL selectively suppresses PRC1 expression in AGS cells at both mRNA and protein levels where p53 was induced concordantly. However, it failed changing PRC1 expression level in HGC27 cells which revealed no p53 induction (Fig. 6C and D). We next determined whether PL suppresses PRC1 gene expression in a p53‐dependent manner by knocking down p53 using siRNA targeting p53 mRNA. As expected, p53 depletion partly attenuated PRC1 inhibition led by PL treatment (10 μM) in AGS cells (Fig. 6E). To further validate the p53‐dependent regulatory role of PL on PRC1, AGS cells were first transfected with different PRC1 promoter‐driven luciferase reporter vectors (as shown in Fig. S3) and TK‐Renilla reporter vector, followed by PL treatment for 24 hrs, and then, the PRC1 promoter activities in transfected cells were determined. Shortening of the PRC1 promoter progressively reduced the luciferase activity of the reporter vectors. Further shortening to 159 bp resulted in a drastic drop in activity which was even barely detected. In addition, PL led to further reduction in the luciferase activity of pGL3‐1744, pGL3‐1504, pGL3‐904, pGL3‐289 and pGL3‐232, respectively (Fig. 6F). Similar results were obtained in AGS and HGC27 cells when transfected with p53‐expressing plasmids or empty vector (Fig. S4A). These data suggested that the major PL‐related functional promoter may located within the region between −232 and −159 bp of the PRC1 promoter, which coincides with the p53‐responsive region identified by Li *et al*. 21 To further elucidate whether PL‐induced p53 interacts with the functional PRC1 promoter *in vivo*, ChIP analysis was conducted in AGS cells treated or untreated with PL using anti‐p53 antibodies followed by quantitative PCR amplification of amplicons that spread across a 4‐kb region of the gene. As shown in Figure S4B, although the signal of p53 at all loci in control cells was very low, there was a remarkable enhancement of p53 signal within the region from −281 to +76 of the PRC1 gene that contained the p53‐responsive region when cells were treated with PL. Concordantly, an amplicon spanning the core promoter of p21 gene was also enriched by the p53 antibody, suggesting the validity of the ChIP assay (Fig. S4B). Collectively, these data suggested that PL targets PRC1 *via* a p53‐dependent manner in gastric cancers.

## Discussion

Gastric cancer is a lethal disease with dismal prognosis, and it can be initiated and driven by a multitude of intrinsic and extrinsic factors 30. Despite the increasing knowledge on risk factors, the molecular mechanisms underlying gastric carcinogenesis have yet been completely deciphered. In this study, we verified that PRC1 is significantly overexpressed in gastric cancers and that its overexpression is strongly associated with poor prognosis of patients. We further demonstrated that PRC1 is essential for the survival of gastric cancer cells. In addition, PRC1 was discovered to be a novel target of PL, which functions as a novel therapeutic agent targeting various types of human malignancies including gastric cancers 17, 31, 32, 33, 34.

PRC1 was originally identified as a CDK substrate in an *in vitro* phosphorylation screen and was subsequently shown to be a mid‐zone‐associated protein required for cytokinesis 9. PRC1 forms oligomers *in vivo* and has microtubule‐binding and microtubule‐bundling activities 9, 10. Perturbing the function of PRC1 or PRC1‐related orthologs in various species inhibits the formation of mid‐zone interdigitating microtubule bundles, resulting in two disarrayed half spindles 10, 35, 36, 37. PRC1 is negatively regulated by p53 and overexpressed in p53‐defective cells 21, suggesting that the gene is tightly regulated in a cancer‐specific manner. However, studies on the functional role of PRC1 in malignancies have been quite limited. Shimo *et al*. first reported that significant overexpression of PRC1 in breast cancer and demonstrated its critical roles in tumour cell growth 12. PRC1 was also found to be up‐regulated in HCC samples and cooperated with Wnt signalling in a positive feedback loop to promote early recurrence 14, 15. Insofar as we know, PRC1 has not been previously implicated in gastric carcinogenesis. To the best of our knowledge, our current study is the first report describing an oncogenic role of PRC1 in regulating the growth and mobility of gastric cancer. This was supported by the infringed proliferation and motility ability by PRC1 inhibition. Gene silencing of PRC1 impaired cytokinesis and led to formation of multi‐nucleated cells in AGS cells. However, depletion of PRC1 gene in HGC27 cells failed in leading to appearance of multi‐nucleated cells. Our data indicated that PRC1 depletion down‐regulated the mitotic‐related genes including KIF4A, BUB1B, AURKB, NEK2, NUSAP1, MELK and TOP2A in AGS instead of HGC27 cells. Therefore, it is likely that PRC1 gene knockdown could contribute to cancer repression *via* mechanisms independent of its cytokinesis‐related function. The precise molecular mechanisms by which PRC1 preferentially modulates multi‐nucleation in AGS cells are under investigation in our laboratory and remain to be elucidated.

Another intriguing finding is the identification of PRC1 as a novel target of PL. PL is a biologically active component from long pepper and has been reported to kill multiple types of cancer cells by targeting the response to reactive oxygen species (ROS) and exerts antitumour activities in a variety of animal models 32, 34, 38, 39. We previously reported that PL significantly suppressed gastric cancer both *in vitro* and *in vivo* by increasing ROS generation and GADD45α expression and decreased the expression of telomerase reverse transcriptase (TERT) gene 17. We now further demonstrated PL also targets PRC1 gene transcriptionally in gastric cancer cells in a functional p53‐dependent manner. Recent genome study has confirmed the high frequency of p53 gene mutations in cancer 40. In gastric carcinomas, p53 gene is mutated in nearly half of the cases 6, 30, and clinical studies have shown that mutant p53‐carrying tumours respond less well to conventional chemotherapeutic drugs and have worse prognosis than wild‐type p53‐carrying tumours 30. Given the high frequency of p53 gene mutations in a wide range of human tumours, the development of efficient mutant p53‐reactivating anticancer drugs has emerged as a promising strategy for improved cancer therapy. Thus far, several small molecules (*i.e*. APR‐246 and ReACp53) which restore wild‐type activity of mutant p53 have been identified using various approaches 41, 42, 43, 44. Further prospective studies are anticipated to identify whether these mutant p53‐reactivating anticancer drugs may function with PL in a synergistic manner to block gastric carcinogenesis.

To summarize, we for the first time report the essential role of microtubule‐associated gene PRC1 in gastric carcinogenesis. Our data should contribute to a better understanding of the molecular mechanism of gastric carcinogenesis and indicate that PRC1 is a promising molecular target for gastric cancer treatment.

## Funding information

This study was supported by National Natural Science Foundation of China (No. 81401974, No. 81472756, No. 81272742 and No. 81401977) and by Natural Science Foundation from the Department of Science &Technology of Jiangsu Province (BK 20140104) and was simultaneously supported by the Fundamental Research Funds for the Central Universities from Nanjing University (No. 20620140709) as well as the Outstanding Youth Project of Nanjing City (JQX15007 and JQX14005).

## Disclosure statement

The authors have no conflict of interest.

## Supporting information


**Figure S1** (A) Comparison of PRC1 mRNA levels between Lauren subtypes in TCGA gastric cancer cohorts. (B) Comparison of PRC1 mRNA levels between the new molecular subtypes in TCGA gastric cancer cohorts. CIN, chromosomal instability; EBV, Epstein‐Barr virus‐positive; GS, genomically stable; MSI, microsatellite instability. (C) Distribution of Lauren subtypes in TCGA cohort with regard to the new molecular classification. (D) Quantification of PRC1 protein expression in gastric cancers and adjacent nontumoral tissues. (E) Kaplan–Meier analysis of overall survival according to PRC1 staining status in gastric adenocarcinoma.

**Figure S2** Ploidy analysis of AGS cells expressing indicated shRNA by flow cytometry analysis.

**Figure S3** Schematic representation of various PRC1 promoter reporters.

**Figure S4** (A) Normalized luciferase activity of various PRC1 promoter reporters in AGS and HGC27 cells transfected with p53‐expressing plasmid (black) or empty vector (white). Results are the mean ± S.D. of triplicate measurements. Data shown are results of a representative from 3 independent experiments. *P < 0.01. (B) ChIP‐qPCR analysis of the abundance of p53 at PRC1 gene in AGS cells treated with piperlongumine at 10 μM or with vehicle control for 24 hrs. Signal of p53 at the p21 gene promoter was used as a positive control. As a negative control, p53 antibody was replaced by IgG (not shown). Values are expressed as % of input. Results represent means ± S.D. from at least three independent experiments *P < 0.05 by Student's *t*‐test.

**Table S1** qRT‐PCR primers.

**Table S2** ChIP‐qPCR primers.

**Table S3** Correlation analysis.
