# Supplementary figures and images for: Elevated PRC1 in gastric carcinoma exerts oncogenic function and is targeted by piperlongumine in a p53‐dependent manner
Source: J Cell Mol Med. 2017 Feb 12;21(7):1329–41. doi: 10.1111/jcmm.13063 (PMC5487922; doi:10.1111/jcmm.13063)

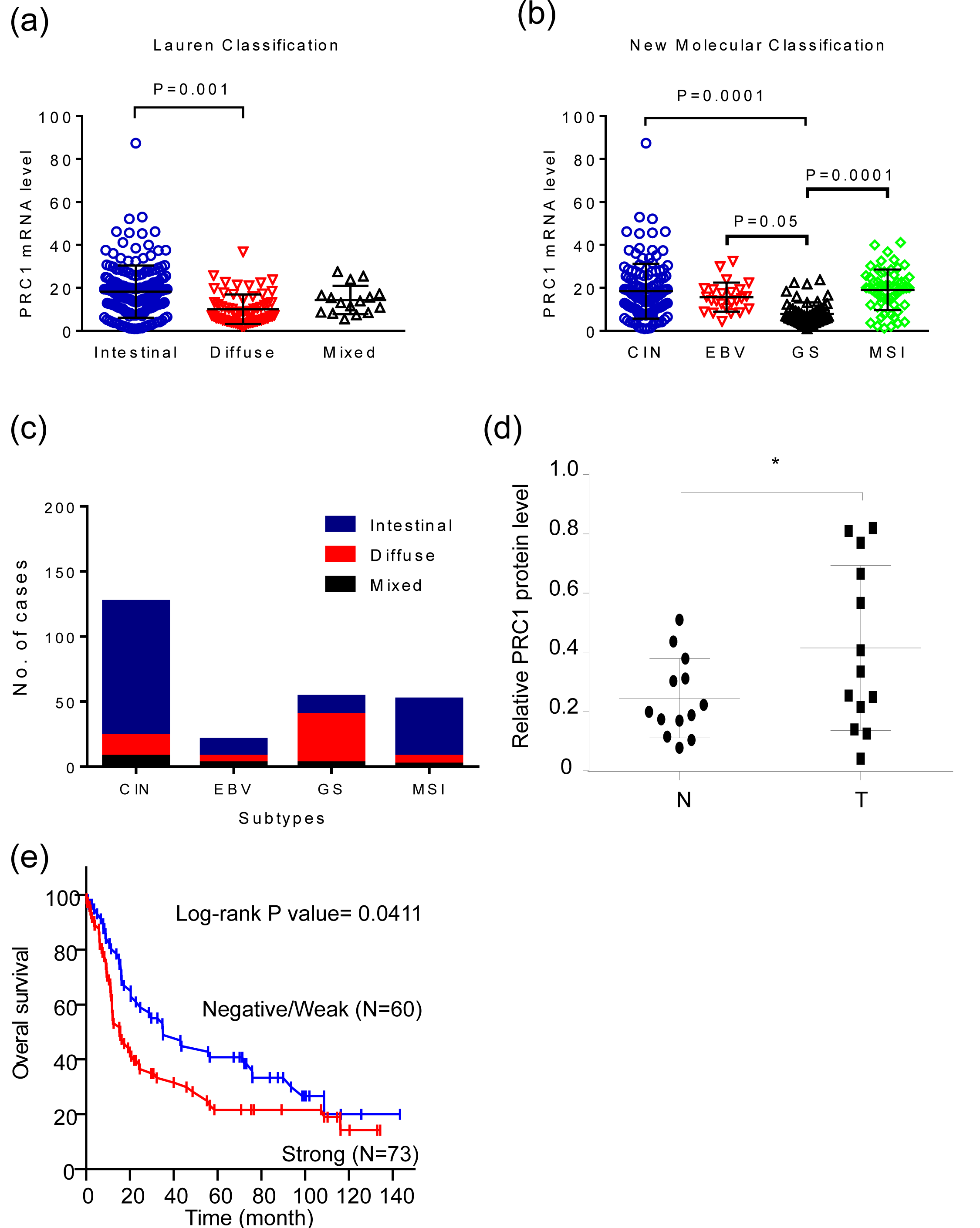

Supplement: Supplementary file 1 — Figure S1 (A) Comparison of PRC1 mRNA levels between Lauren subtypes in TCGA gastric cancer cohorts. (B) Comparison of PRC1 mRNA levels between the new molecular subtypes in TCGA gastric cancer cohorts. CIN, chromosomal instability; EBV, Epstein‐Barr virus‐positive; GS, genomically stable; MSI, microsatellite instability. (C) Distribution of Lauren subtypes in TCGA cohort with regard to the new molecular classification. (D) Quantification of PRC1 protein expression in gastric cancers and adjacent nontumoral tissues. (E) Kaplan–Meier analysis of overall survival according to PRC1 staining status in gastric adenocarcinoma. [file JCMM-21-1329-s001.tif]

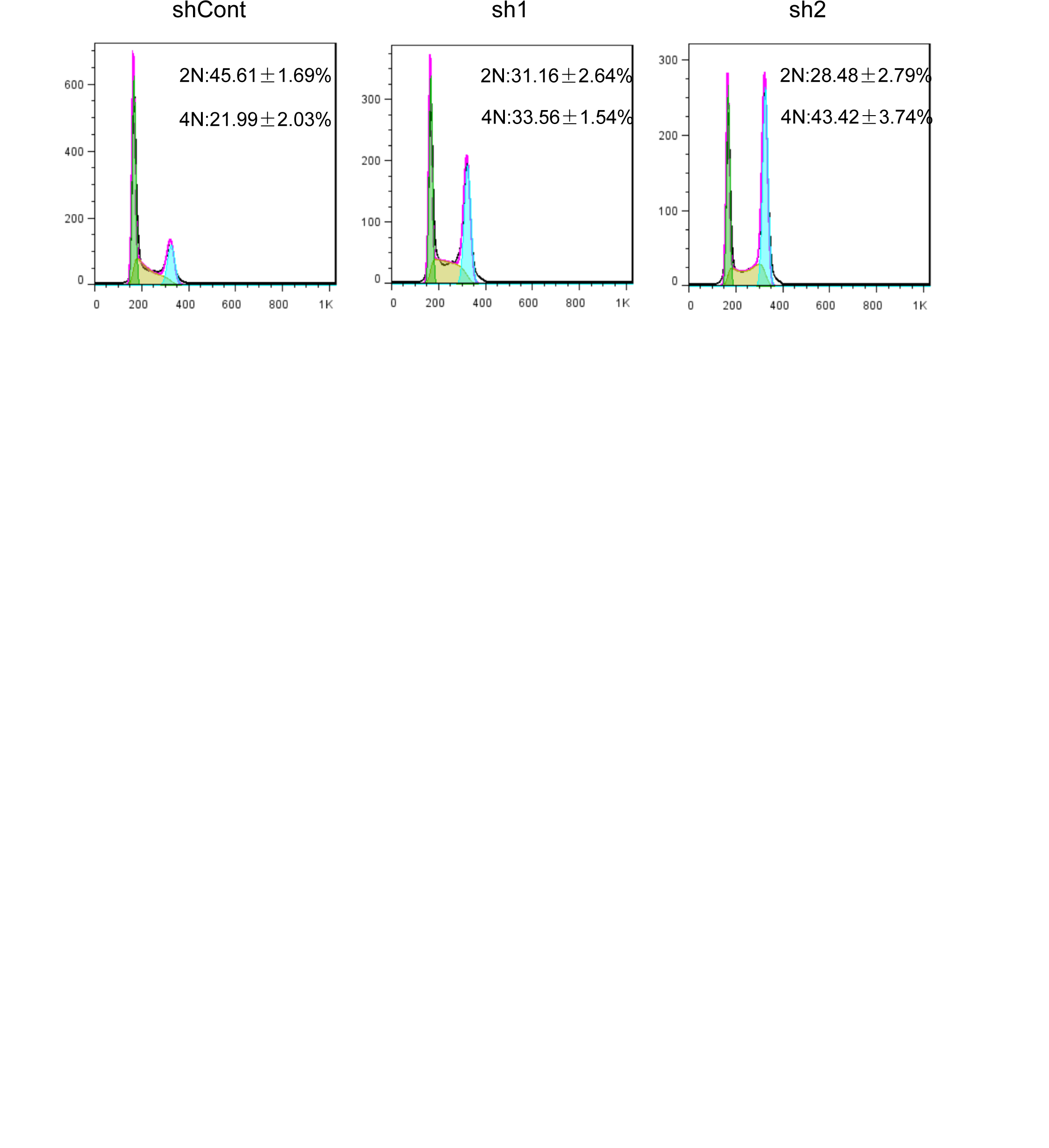

Supplement: Supplementary file 2 — Figure S2 Ploidy analysis of AGS cells expressing indicated shRNA by flow cytometry analysis. [file JCMM-21-1329-s002.tif]

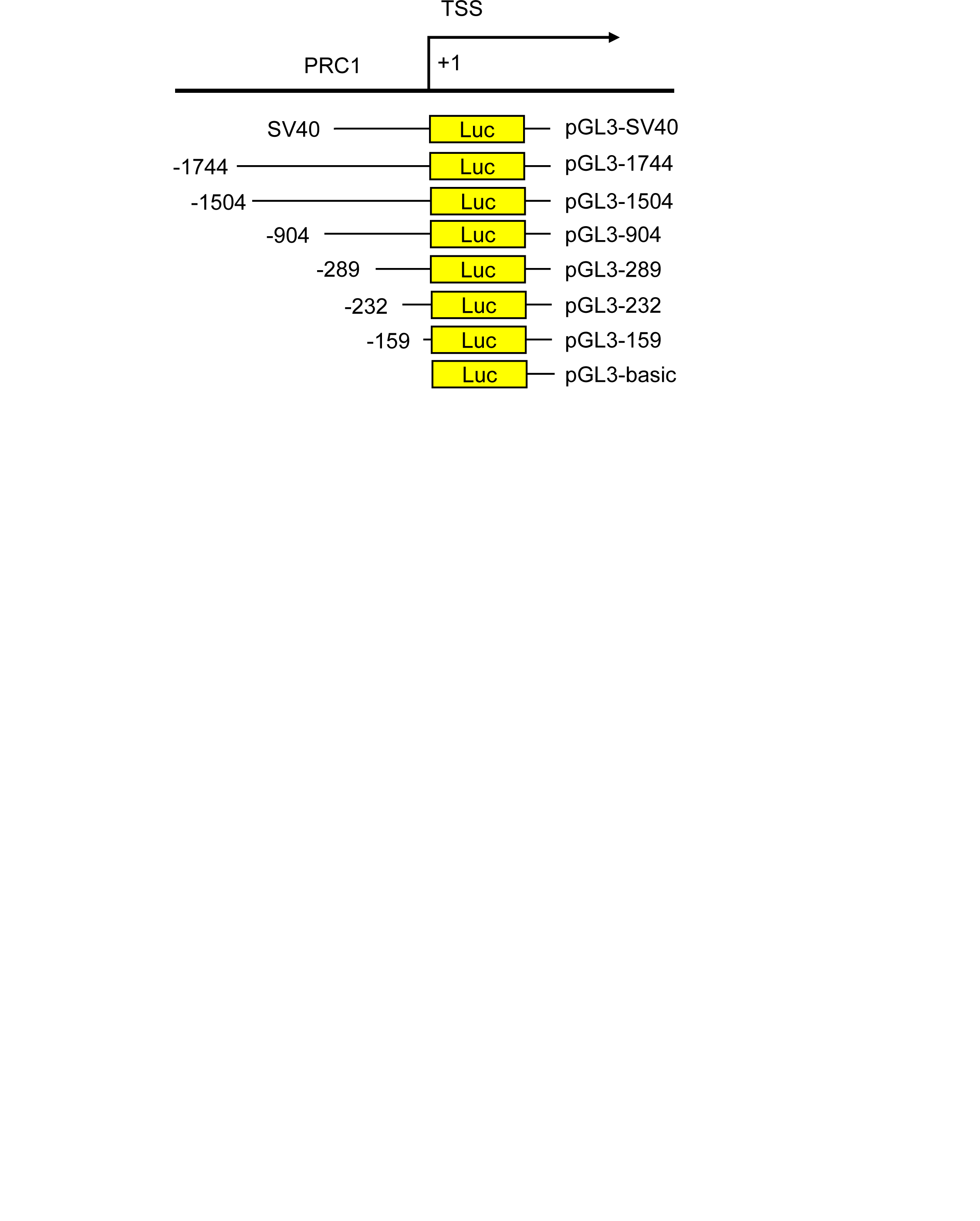

Supplement: Supplementary file 3 — Figure S3 Schematic representation of various PRC1 promoter reporters. [file JCMM-21-1329-s003.tif]

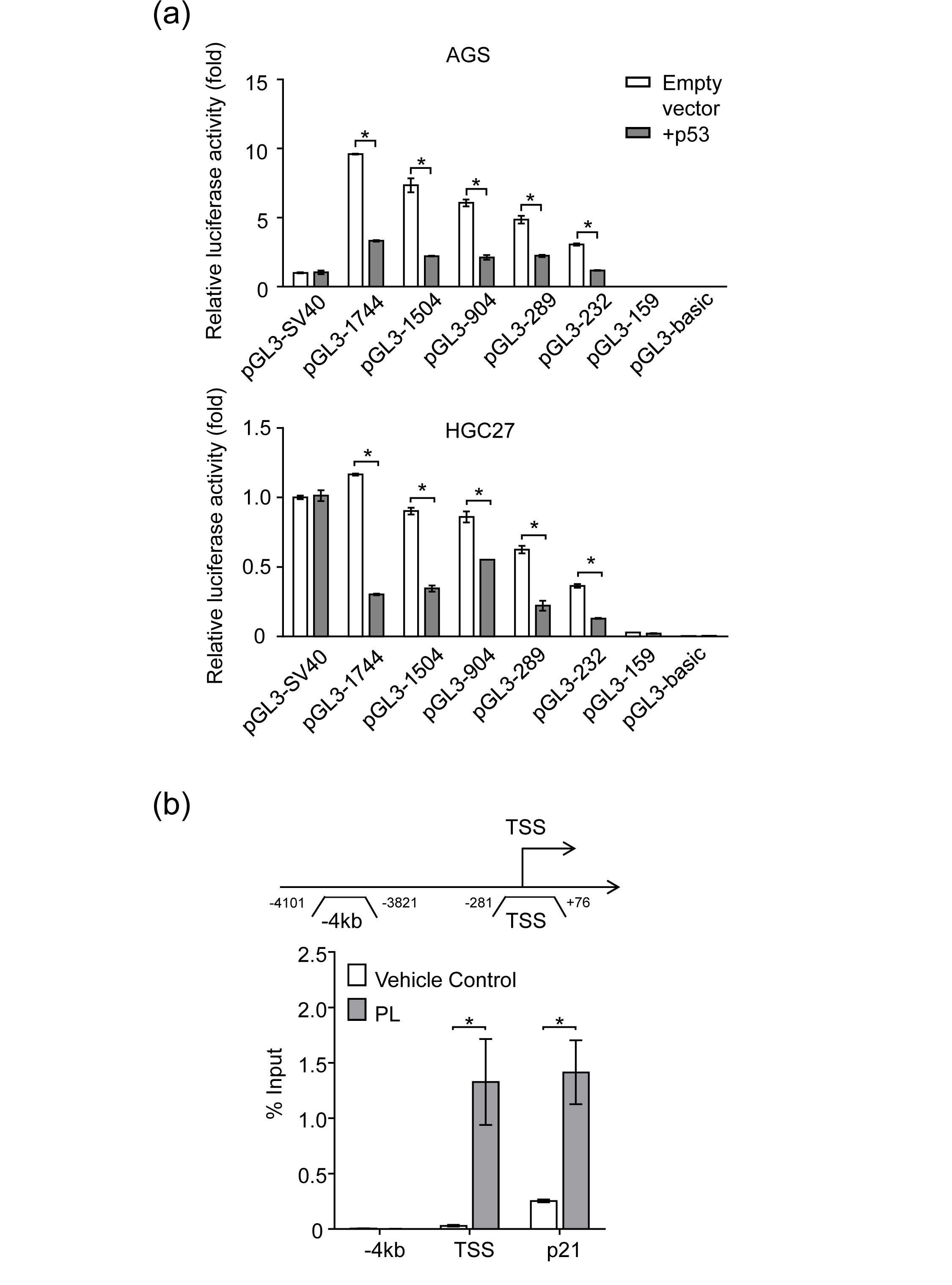

Supplement: Supplementary file 4 — Figure S4 (A) Normalized luciferase activity of various PRC1 promoter reporters in AGS and HGC27 cells transfected with p53‐expressing plasmid (black) or empty vector (white). Results are the mean ± S.D. of triplicate measurements. Data shown are results of a representative from 3 independent experiments. *P < 0.01. (B) ChIP‐qPCR analysis of the abundance of p53 at PRC1 gene in AGS cells treated with piperlongumine at 10 μM or with vehicle control for 24 hrs. Signal of p53 at the p21 gene promoter was used as a positive control. As a negative control, p53 antibody was replaced by IgG (not shown). Values are expressed as % of input. Results represent means ± S.D. from at least three independent experiments *P < 0.05 by Student's t‐test. [file JCMM-21-1329-s004.tif]
