# Supplementary material for: Elevated PRC1 in gastric carcinoma exerts oncogenic function and is targeted by piperlongumine in a p53‐dependent manner
Source: J Cell Mol Med. 2017 Feb 12;21(7):1329–41. doi: 10.1111/jcmm.13063 (PMC5487922; doi:10.1111/jcmm.13063)
Supplement: Supplementary file 5 — Table S1 qRT‐PCR primers. [file JCMM-21-1329-s005.docx]

| Supplementary Table 1:The sequences of primers for real time RT-PCR | | |  |
| --- | --- | --- | --- |
| Gene | Strand | Primer sequence 5’ to 3’ | Size(bp) |
| β-actin | Forward  Reverse | CTCTTCCAGCCTTCCTTCCT  AGCACTGTGTTGGCGTACAG | 116 |
| PRC1 | Forward  Reverse | TAGACCACACCCCAGACACA  GTGGCCACAGCTTCTCTTTC | 223 |
| KIF4A | Forward  Reverse | TACTGCGGTGGAGCAAGAAG  CATCTGCGCTTGACGGAGAG | 100 |
| BUBIB | Forward  Reverse | AAATGACCCTCTGGATGTTTGG  GCATAAACGCCCTAATTTAAGCC | 184 |
| AURKB | Forward  Reverse | CAGAAGAGCTGCACATTTGACG  CCTTGAGCCCTAAGAGCAGATTT | 136 |
| NEK2 | Forward  Reverse | TGCTTCGTGAACTGAAACATCC  CCAGAGTCAACTGAGTCATCACT | 195 |
| NUSAP1 | Forward  Reverse | AGCCCATCAATAAGGGAGGG  ACCTGACACCCGTTTTAGCTG | 186 |
| MELK | Forward  Reverse | TATTCACCTCGATGATGATTGCG  AGAAAGCCTTAAACGAACTGGTT | 169 |
| TOP2A | Forward  Reverse | ACCATTGCAGCCTGTAAATGA  GGGCGGAGCAAAATATGTTCC | 129 |
