# Supplementary material for: Elevated PRC1 in gastric carcinoma exerts oncogenic function and is targeted by piperlongumine in a p53‐dependent manner
Source: J Cell Mol Med. 2017 Feb 12;21(7):1329–41. doi: 10.1111/jcmm.13063 (PMC5487922; doi:10.1111/jcmm.13063)
Supplement: Supplementary file 6 — Table S2 ChIP‐qPCR primers. [file JCMM-21-1329-s006.docx]

| Supplementary Table 2: The sequences of primers for ChIP-qPCR | | | |
| --- | --- | --- | --- |
| Amplicon | Strand | Primer sequence 5’ to 3’ | Size(bp) |
| -4kb | Forward  Reverse | TGCTTCTTGGCCTGGACTTT GCTGATGCCTGAAATGGCTC | 281 |
| TSS | Forward  Reverse | CTTGAGGCTGCCGCCAAGCCAG CGGACGCTCCAAGCAGCCGTGAG | 357 |
| p21 | Forward  Reverse | GTGGCTCTGATTGGCTTTCTG CTGAAAACAGGCAGCCCAAG | 105 |
