# Supplementary material for: Elevated PRC1 in gastric carcinoma exerts oncogenic function and is targeted by piperlongumine in a p53‐dependent manner
Source: J Cell Mol Med. 2017 Feb 12;21(7):1329–41. doi: 10.1111/jcmm.13063 (PMC5487922; doi:10.1111/jcmm.13063)
Supplement: Supplementary file 7 — Table S3 Correlation analysis. [file JCMM-21-1329-s007.docx]

|  | Nagative/Weak | Strong | *P*-value |
| --- | --- | --- | --- |
| Gender |  |  |  |
| Male | 39 | 46 | 0.8124 |
| Female | 21 | 27 |  |
| Age (years) |  |  |  |
| ≤60 | 19 | 18 | 0.3694 |
| >60 | 41 | 55 |  |
| Grade |  |  |  |
| 1 | 4 | 2 | 0.5377 |
| 2 | 24 | 32 |  |
| 3 | 32 | 39 |  |
| Stage |  |  |  |
| 1 | 9 | 8 | 0.8287 |
| 2 | 8 | 9 |  |
| 3 | 24 | 28 |  |
| 4 | 19 | 28 |  |
| Stage (T) |  |  |  |
| 1 | 2 | 5 | 0.5073 |
| 2 | 19 | 17 |  |
| 3 | 35 | 48 |  |
| 4 | 4 | 3 |  |
| Stage (N) |  |  |  |
| 0 | 10 | 8 | 0.3724 |
| 1 | 15 | 21 |  |
| 2 | 24 | 23 |  |
| 3 | 11 | 21 |  |
| Stage (M) |  |  |  |
| 0 | 51 | 62 | 0.9912 |
| 1 | 9 | 11 |  |
| Lymph node | |  |  |
| Negative | 10 | 8 | 0.3383 |
| Positive | 50 | 65 |  |
| *H. pylori* |  |  |  |
| Absence | 19 | 23 | 0.9629 |
| Presence | 37 | 44 |  |
| Type |  |  |  |
| Intestinal | 35 | 48 | 0.2206 |
| Diffuse | 19 | 14 |  |
| Mixed | 6 | 11 |  |

Supplementary Table 3: Correlation of PRC1 expression with clinicopathological features
